# The intestinal epithelium tuft cells: specification and function

**DOI:** 10.1007/s00018-012-0984-7

**Published:** 2012-04-19

**Authors:** François Gerbe, Catherine Legraverend, Philippe Jay

**Affiliations:** 1 CNRS UMR5203, 34094 Montpellier, France; 2 INSERM U661, 34094 Montpellier, France; 3 Montpellier I University, 34094 Montpellier, France; 4 Montpellier II University, 34094 Montpellier, France; 5 Institute of Functional Genomics, 141 rue de la Cardonille, 34094 Montpellier Cedex 5, France

**Keywords:** Tuft cells, Brush cells, Intestinal epithelium, Atoh1, Cell differentiation, Dclk1

## Abstract

The intestinal epithelium, composed of at least seven differentiated cell types, represents an extraordinary model to understand the details of multi-lineage differentiation, a question that is highly relevant in developmental biology as well as for clinical applications. This review focuses on intestinal epithelial tuft cells that have been acknowledged as a separate entity for more than 60 years but whose function remains a mystery. We discuss what is currently known about the molecular basis of tuft cell fate and differentiation and why elucidating tuft cell function has been so difficult. Finally, we summarize the current hypotheses on their potential involvement in diseases of the gastro-intestinal tract.

## Scope of this review

During the five decades that followed their discovery in the rat trachea [1] and in the mouse glandular stomach [2], the characterization of tuft cells has been hampered by the lack of specific molecular markers, and next to nothing was known about their functions, but this is changing. Several molecular markers that allow their unambiguous identification are now available, and these can be used to create mouse lines in which reporters are specifically expressed in tuft cells. These advances are making tuft cells amenable to thorough analysis, and we anticipate that insights into their functions will be obtained in the near future. The ultrastructural properties of tuft cells have already been the subject of excellent reviews [3, 4] and will not be detailed here. We will instead summarize recently published data on their protein expression profile, developmental timing, and lineage relationships to the other cell types composing the intestinal epithelium. We will also discuss their potential implication in intestinal diseases, and, relying on data from other organs, speculate on how they may contribute to intestinal homeostasis.

## Cellular composition of the small intestinal epithelium

**Fig. 1:**
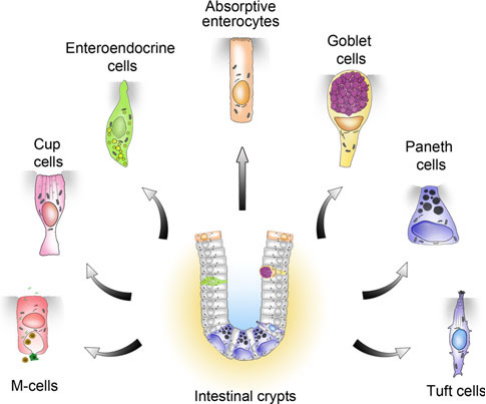
Schematic representation of the known intestinal epithelial cell types generated from Lgr5-expressing crypt base columnar stem cells

The intestinal epithelium is composed of a folded cell monolayer organized into crypts that invaginate into the underlying mesenchyme, and villi projecting into the lumen. The crypts and villi comprise the proliferative and differentiated compartments, respectively. Each villus receives inputs from several crypts and is therefore polyclonal. The stem cells, on which the rapid and permanent renewal of the intestinal epithelium relies, are located in the base of the crypts, yet their precise nature is still a matter of debate [5]. The progeny of the stem cell population feeds into shorter-lived progenitors of the so-called “transit-amplifying” zone while moving upwards. Migration continues but proliferation ceases when cells reach the crypt–villus boundary, and the villus contains only post-mitotic cells. Although at least seven different cell types have now been identified in the intestinal epithelium (Fig. 1), only four are usually considered, and several lineage analyses have shown that these four cell types are produced from the crypt base columnar stem cells [6–8]. Enterocytes are responsible for nutrient absorption and represent the vast majority of villous cells in the small intestine. Goblet cells are scattered throughout the epithelium and produce a protective mucus layer. Hormone-producing enteroendocrine cells represent approximately 1 % of all epithelial cells, and regulate various functions of the intestinal epithelium, and beyond. Paneth cells, clustered in the bottom of the crypts, produce antimicrobial peptides that regulate the gut microbiota, as well as growth factors involved in the maintenance of the neighboring stem cells, and in the healthy mouse, their presence is limited to the small intestine. Whereas the entire epithelium is renewed within 4–7 days, Paneth cells have a life span of about 2 months. Except for the absence of villi, the more numerous goblet cells, and the absence of Paneth cells in the crypts, the architecture of the colon closely resembles that of the small intestine.

Although they are usually overlooked, at least three additional cell types exist in the intestinal epithelium: microfold or “membranous” (M) cells, cup cells, and tuft cells. M cells cover the surface of the gut-associated lymphoid follicles and function as an interface between the luminal content and the underlying immune cells [9]. The wine glass-shaped cup cells comprise up to 6 % of the epithelial cells of the ileum and therefore surpass enteroendocrine cells in frequency. Cup cells are characterized by a shorter brush border with linear arrays of particles in their microvillous membrane, and a markedly weaker alkaline phosphatase activity than that of other columnar cells [10]. Like M cells, cup cells express vimentin [11], but they differ from M cells in the glycosylation pattern of their plasma membrane and do not transport antigens and pathogens to mucosal immune cells [12]. Last but not least, tuft cells constitute a minor fraction (0.4 %) of the adult mouse intestinal epithelium, but like cup cells their precise functions are still unknown.

## History of tuft cell discovery

The identification of tuft cells as a distinct entity emerged after several independent studies reported the presence of unusual epithelial cell types in various hollow organs. The first observations are usually attributed to Rhodin and Dalhamn, who described cells with a well-developed apical brush border in the rat trachea [1], and Järvi and Keyrilainen who found similar cells in the mouse glandular stomach [2]. The presence of such cells was later confirmed in the respiratory tract [13–18] and the GI tract [19–28], of many mammals including human, cat, pig, cow, rat, mouse, rabbit, guinea pig, and ferret [29].

Depending on which morphological criterion was retained, they were named “peculiar”, “fibrillovesicular”, “caveolated”, “brush”, or “tuft” cells, all appellations referring to epithelial cells endowed with a unique tubulovesicular system and apical bundle of microfilaments connected to a tuft of long and thick microvilli protruding into the lumen. Although tuft cells may exert different functions depending on their location, most researchers now agree that they represent a particular epithelial cell type. Following the recommendation made by a working group in 2005 [29], we will use the name “tuft” cells throughout this review.

## Reliable and unreliable markers: clues to the functions of tuft cells?

Within the hollow organs in which they reside, tuft cells from the airway and digestive apparatus display unique morphological features and express signaling components typical of chemosensory cell types, yet it is still unclear to what extent their physiological roles are related. For clarity, this survey of tuft cell markers (summarized in Table 1) is organized according to their nature.

**Table 1:** Overview of the proposed tuft cell markers

	Immunoreactivity in tuft cells	Immunoreactivity in other epithelial cells
Structural markers		
Villin	Strong at the apex, weaker at the basolateral membrane [18]	Enterocytes brush border [18]
Fimbrin	Strong at the apex, weaker at the basolateral membrane [18]	Enterocytes brush border [18]
α- or β-tubulin	Highlights the dense microtubule network [30]	All epithelial cells [30]
Ac-tubulin	Highlights the dense apical microtubule network [35]	
Ankyrin	Strong at the basolateral membrane [30]	Enterocytes basolateral membrane [30]
CK-18	Cytoplasmic [30]	–
Neurofilaments	Supranuclear cytoplasmic [32]	
Dclk1	Highlights the dense microtubule network [34]	Subset of enteroendocrine cells [64], putative quiescent stem cells [71]
Taste cell-related markers		
α-gustducin	Faint basolateral staining with strong apical immunoreactivity (rat duodenum) or whole cell staining (mouse small intestine) [36, 46]	Expressed in K, L, or K/L enteroendocrine cells [44–46]
Trpm5	Strong at the basolateral membrane [39]	Enteroendocrine cells [38]
T1R1/T1R3	Apical side of the cell [38]	Secretory granules of Paneth cell, brush border of enterocytes and enteroendocrine cells [44, 47, 49]
Other markers		
Ptgs1, Ptgs2	Cytoplasmic [34, 40]	Strongly inducible during inflammation
H-Pgds	Whole cell [34]	–
UEA1 lectin	Apical side of the cell [54]	Enteroendocrine, goblet and Paneth cells [46, 56, 57]
Sox9	Nuclear [34]	Paneth, stem/progenitor cells [58–60]. eGFP-positive enteroendocrine cells in a Sox9-eGFP reporter strain [61]
L-FABP	Cytoplasmic [62]	Enteroendocrine cells [63]
NA^+^/K^+^ ATPase	Cytoplasmic [50, 51]	

## Structural markers

**Fig. 2:**
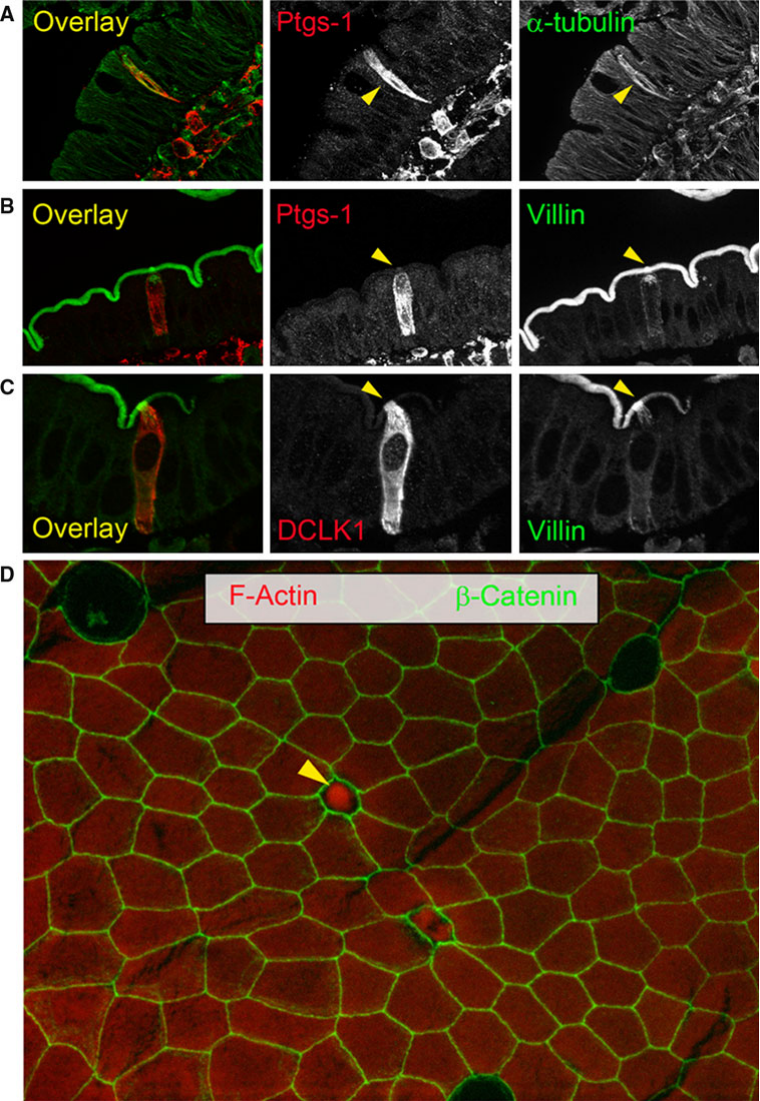
Unique molecular and structural features distinguish tuft cells from the other intestinal epithelial cells. **a**–**d** Tuft cells are indicated by *arrowheads*. **a** α-tubulin staining (*green*) highlights the dense microtubule network of a tuft cell co-stained for Ptgs-1 (*red*). **b** Tuft cell co-stained for Ptgs-1 (*red*) and villin (*green*). **c** Tuft cell co-stained for DCLK1 (*red*) and villin (*green*). Note the increased Villin immunoreactivity of the apical pole and rootlets (*yellow* *arrowhead*). **d** Surface epithelium of a villus (whole mount) stained for β-catenin (*green*) and phalloidin (*red*). The apical part of a tuft cell highly immunoreactive for F-actin sticks out into the lumen (*yellow* *arrowhead*)

The first molecular markers used for tuft cell recognition were related to their unique ultrastructural features (Fig. 2). Antibodies against actin filament cross-linking proteins such as villin and fimbrin were shown to react strongly with the apex, and to a lesser extent, with the basolateral cytoplasm of tuft cells [18]. In addition, staining of the tubulin network or of members of the ankyrin family of adaptor proteins was later proposed as a means of discriminating between tuft cells and other cell types [30]. However, several of the above-mentioned markers are shared between intestinal tuft cells and enterocytes, and are better suited for the identification of tuft cells in the airways, and the gastric and pancreatic duct epithelia. In contrast, the network of cytokeratin 18 (CK-18) filaments extending from the cell periphery to the perinuclear region, and the association of neurofilaments [31, 32], actin filaments, acetylated form of tubulin (ac-tubulin), and microtubule-linked protein kinase Dclk1 (also known as Dcamkl-1), are much more restricted to tuft cells [33–35]. The combination of Dclk1, CK-18, and neurofilaments is not known to occur in other cell types.

## Taste cell-related markers

Right from the start, and based on structural similarities with lingual taste bud cells, tuft cells have been suspected to be involved in chemoreception. This assumption was strengthened when α-gustducin and other members of the taste transduction pathway were detected in tuft cells of the intestine and the pancreatic duct [28, 36]. Together with signaling molecules such as β-endorphin, Met-Enkephalin and uroguanylin, tuft cells express the transient receptor potential cation channel, subfamily M, member 5 (TRPM5) [37–39], which transduces signals from bitter-, sweet-, and umami-tasting substances in lingual taste cells. Taking advantage of a Trpm5-GFP reporter mouse, Bezençon and colleagues showed that the GFP-positive intestinal epithelial cells express the taste receptors T1R1 + T1R3 heterodimers that sense amino acid and umami molecules [38, 40] (reviewed in [41, 42]). Furthermore, a *Trpm5*-dependent luminal secretion of β-endorphin was detected following exposure of the mouse duodenum mucosa to hypertonic saline and glucose [43]. Subtle differences in the downstream effectors of the taste transduction pathway, illustrated by the expression of Phospholipase C-β-2 (PLCβ2) in gastric tuft cells [35], and PLCγ2 in tuft cells of the intestinal epithelium [38, 40], suggest potentially distinct, yet chemoreception-related functions for these cells.

The taste cell-related markers should, however, be used with caution. Firstly, whereas all CK-18-positive tuft cells express α-gustducin and Trmp5, a subset of Trpm5^+^ α-gustducin^+^ cells was found to be CK-18-negative [39], including enteroendocrine cells that secrete GLP-1/GIP (also known as L, K, or K/L cell subtypes) or serotonin [40–46]. Secondly, a positive signal for T1R1 and T1R3 was detected in granules of Paneth cells, and in the apical membrane of enterocytes and enteroendocrine cells [44, 47–49].

Taken together, these studies indicate that taste transduction-related proteins, although indicative of a chemosensory function, should not be considered as specific markers of tuft cells.

## Markers of the eicosanoid biosynthesis pathway

Several members of the eicosanoid pathway are listed in the microarray data derived from mouse intestinal epithelial Trpm5-eGFP cells, suggesting a role played by tuft cells in the modulation of intestinal smooth muscle contraction [40]. In normal conditions, tuft cells are also the only epithelial cells that constitutively express all the enzymes necessary for prostaglandin-D_2_ (PGD2) biosynthesis, including the hematopoietic prostaglandin-D synthase (HpgDs) and the prostaglandin-endoperoxide synthases, Ptgs1 and Ptgs2 [34, 40]. However, the target cells that express the cognate receptors have not been identified.

## Other markers

Angiotensinogen, renin, and succinate receptor genes are listed in the microarray data derived from mouse intestinal epithelial Trpm5-eGFP cells, suggesting that a role is played by tuft cells in the regulation of water and sodium transport, vasomotricity, and blood pressure [40]. Like the mitochondria-rich chloride cells found in fish gills and bird nasal glands, tuft cells strongly express the Na^+^/K^+^ ATPase, involved in electrolyte secretion and absorption, as well as other proteins that are mainly involved in NaHCO_3_ secretion [50, 51]. The fact that tuft cells are over-represented (>30 %) in the gastric groove, and respond within minutes to tetragastrin stimulation by secreting an alkaline solution (probably NaHCO3), may serve to neutralize HCl and to protect the squamous epithelium of the forestomach and the esophagus [52]. Likewise, the relatively high percentage (>2 %) of tuft cells in the proximal duodenum and in the cecum, may function to limit the damage to the mucosa caused by acidic food coming from the stomach, or organic acids produced by bacteria [52]. Whether tuft cells scattered throughout the rest of the intestinal mucosa serve similar or totally different physiological functions remains to be determined. In this connection, it is important to note that nitric oxide synthase (nNOS) and NADP-linked glucose-6-phosphate dehydrogenase were found to be expressed in tuft cells of the stomach [53], but are absent from intestinal tuft cells [46].

Several studies have revealed an increased reactivity of the fucose-reactive Ulex europaeus agglutinin 1 (UEA-1) lectin towards the tuft cell glycocalyx [54, 55]. However, it should be noted that UEA1 binding capacity has also been reported for other cell lineages including enteroendocrine cells [46, 56], M cells of the Peyer patches [57], as well as for the secretory granules of Paneth cell and mucous-containing vacuoles of goblet cells. Similarly, in addition to being strongly expressed in tuft cells [34], the Sox9 transcription factor is expressed in Paneth cells and in stem and progenitor cells [58–60], and might also be expressed in some enteroendocrine cells [46, 53, 61]. Proteins involved in fatty acid metabolism have also been shown to be expressed in tuft cells. Of particular note, the liver isoform of fatty acid-binding protein-1 (L-FABP) is strongly expressed in tuft cells of the stomach, common bile duct, and large intestine of the adult rat [62, 63], suggesting that tuft cells may be involved in fatty acid sensing or absorption. However, type D enteroendocrine cells also stain positive for L-FABP [50, 51, 63].

Last but not least, the zinc-finger transcriptional repressor Gfi1b was recently detected in nuclei of tuft cells and no other epithelial cells [64]. Gfi1b is related to Gfi1, known to stabilize the goblet and Paneth cell intestinal lineages [65] by repressing the pro-endocrine *Neurog3* gene [66].

In conclusion, it is now possible to identify tuft cells based on gene and protein expression, yet unambiguous identification requires a combination of molecular markers, ideally in association with tuft cell-specific morphological features [34, 64]. In addition, these molecular makers should be selected with caution, since some phenotypic heterogeneity exists (i.e., expression of the PLCβ2 isoform into the gastric epithelium and the PLCγ2 within the intestine), that may reflect the existence of different tuft cell sub-types according to their location. From the classes of proteins and surface receptors they express, we speculate that within the normal intestinal epithelium, tuft cells may modulate diverse functions such as chemoreception, differentiation, migration, inflammation, and other integrated physiological responses.

## Development of tuft cells

Although tuft cells appear relatively late in embryonic development, there is no consensus on the precise timing of tuft cell differentiation in the mammalian gut. This probably reflects differences along the proximo-distal axis of the GI tract, as well as the asynchronous onset of expression of the markers used in different studies. In the mouse, Dclk1 expression is first detected in tuft cells of the intestine 1 week after birth [34]. In the stomach and most proximal part of the small intestine, ac-tubulin-positive tuft cells are present as early as E16.5 [35], and Gfi1b-expressing tuft cells are found in the proximal small intestine of E18.5 Gfi1b-eGFP mouse embryos [64]. In the rat, tuft cells can be detected in the stomach after birth and increase in number during a period corresponding to the end of the suckling period [62]. In humans, tuft cells have been identified morphologically in the small intestine of a 5-month-old fetus [67], yet their Dclk1 and ac-tubulin status were not evaluated.

## Cellular origin of tuft cells

The first evidence in favor of the presence of tuft cell progenitors in the crypt came from the observation that the first tuft cells to become labeled after injecting mice with 3H-thymidine appeared in the lower portion of the crypt [24]. The results of a mutagenesis-based clonal analysis later suggested that, together with enterocytes and goblet cells, tuft cells originate from a common progenitor or stem cell [7]. BrdU incorporation studies confirmed that tuft cells are post-mitotic cells that are continuously renewed and have a life span of at least 1 week [34]. Finally, genetic tracing experiments using a cross of the Lgr5^EGFP-IRES-CreERT2^ mouse [6] with the Cre-activable Rosa26-LacZ reporter mouse [68], led to the conclusion that, like enterocytes, enteroendocrine, Paneth and goblet cells, tuft cells originate from Lgr5-expressing crypt base columnar stem cells [34] and this was recently confirmed in organoids derived from single *Lgr5*-*EGFP* cells [69]. Of note, the demonstration of Dclk1 expression in post-mitotic tuft cells in the intestinal epithelium [33] casted serious doubt on the notion that Dclk1-expressing cells are long-lived quiescent stem cells [70, 71].

## Genetic requirements for tuft cell differentiation

**Fig. 3:**
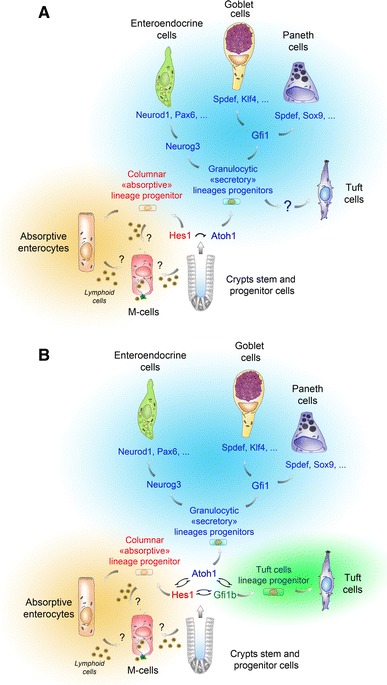
Two different schemes for tuft cell specification and differentiation, and their relationship to the other lineages. Stem cells from intestinal crypts can give rise to at least six known cell types [89]. Whether differentiation of M cells occurs directly from a progenitor or an enterocyte, and requires contacts with lymphocytes remains unclear. In model **a**, tuft cell specification, but not terminal differentiation, is proposed to rely on Atoh1 function. The cell fate of the stem cell progeny relies on Delta-Notch mediated lateral inhibition, leading Hes1 expressing progenitors to adopt an “absorptive” identity and Atoh1 expressing progenitors to adopt a “secretory” (granulocytic) fate. According to subsequent genetic events, Atoh1-expressing progenitors then give rise to mature enteroendocrine, goblet, Paneth or tuft cells. *Neurog3* expression primes cells towards an endocrine program (Neurod1, Pax6) that results in different mature enteroendocrine subtypes. *Gfi1* expression prevents ectopic *Neurog3* expression in Paneth and Goblet cells. To reach a fully mature state, Paneth cells depend on the expression of *Sox9* and *Spdef*, and goblet cells depend on that of *Klf4* and *Spdef*. In model **b**, the cell fate does not rely on two but three transcription factors (Hes1, Atoh1 and Gfi1b), reciprocally antagonizing themselves. As in model **a**, Hes1 expression drives progenitors towards an absorptive cell identity, Atoh1 expression is essential for enteroendocrine, goblet and Paneth cell specification and survival, and Atoh1 function is not required for [64] tuft cell differentiation. The Gfi1b transcription factor, expressed in immature and terminally differentiated tuft cells, is proposed as a molecular switch towards the tuft cell lineage

As already mentioned, the dearth of specific markers and the rarity of tuft cells (0.4 % of epithelial cells) probably explains why, up until very recently, tuft cells have been systematically overlooked in the genetic mouse models used to analyze the regulation of patterning and differentiation of the intestinal epithelium. Such models have, however, proven to be very useful in identifying the differentiation program of the four best-known cell types of the intestinal epithelium. It is now well established that the Wnt and Notch signaling pathway activities are not only required for cell proliferation [72, 73] but also intervene in early cell-fate decisions within the crypt. For instance, interfering with the Wnt pathway by overexpressing the Dickkopf1 inhibitor resulted in the depletion of the goblet, enteroendocrine, and Paneth cells [74], and deletion of Frizzled-5, one of the Wnt receptors, produced immature Paneth cells [75]. Similarly, Delta-Notch-mediated lateral inhibition is involved in the choice of progenitors between absorptive and secretory fates. In cells expressing high levels of the Notch Delta-like ligands Dll1 and Dll4, this process results in repression of the Notch target gene *Hes1* and the ensuing de-repression of the *Atonal homolog 1* (*Atoh1*) gene, encoding a basic helix–loop–helix transcription factor that dictates a secretory fate [76–78]. Among the other genes involved in secretory cell specification (reviewed in [79] and Fig. 3), *Neurogenin 3* (*Neurog3*) encodes a transcription factor, whose deletion results in the complete extinction of the enteroendocrine lineage [80]. Likewise, Paneth cells are totally absent in Sox9 knockout mice [58, 60], and goblet cells are reduced by 90 % in Klf4 knockout mice [81].

Since the recent discovery of new tuft cell markers, it has become possible to use some of the above-mentioned genetically modified mouse lines to explore the lineage relationships between tuft cells and the other epithelial cell types, as well as the genetic requirements for tuft cell differentiation. Because tuft cells are known to produce and secrete opioids [43], the possibility that *Atoh1* expression is required for their specification and/or differentiation, as for the known secretory cell types, was investigated. Indeed, when *Atoh1* ^*LoxP/LoxP*^ *; VillinCre* ^*ERT2*^ mice were submitted to a single daily i.p. injection of tamoxifen (50 mg/kg) for four consecutive days, tuft cells disappeared from the intestinal epithelium, as did goblet, enteroendocrine and Paneth cells [34] (Fig. 3a). Consistent with a role for *Atoh1* in tuft cell specification and/or differentiation, inhibition of Notch signaling was recently shown to result in increased numbers of tuft cells [82]. Surprisingly, therefore, when *Atoh1* deletion was induced in *Atoh1* ^*LoxP/LoxP*^ *; Rosa26* ^*CreERT2*^ mice by 3 daily gavages of tamoxifen (200 mg/kg), tuft cells were present and even over-represented, even though goblet, enteroendocrine and Paneth cells were absent [64]. The potentially higher toxicity of intraperitoneally injected tamoxifen, compared to tamoxifen given *per os*, was proposed as an explanation to resolve this paradox [64]. However, a tamoxifen- or Cre recombinase-mediated toxic effect that would be directed specifically towards tuft cells, and requiring the *Atoh1*-deficient genetic background to manifest itself, remains to be formally demonstrated.

The floxed *Atoh1* alleles were efficiently recombined and the Atoh1 protein was undetectable in FACS-sorted mature tuft cells of *Atoh1* ^*LoxP/LoxP*^ *; Rosa26* ^*CreERT2*^ mice, consistent with a lack of *Atoh1* requirement for differentiation of tuft cells [64]. However, the same study reports a weak EGFP signal in some immature tuft cells in the lower crypt of Atoh1-EGFP reporter mice. Furthermore, a non-negligible fraction (14 %) of the tuft cell population, not compatible with leaky background recombination, was β-Galactosidase-positive in the intestines of *Atoh1*-*Cre* ^*ERT2*^ *; Rosa26*-*LacZ* mice, which was interpreted as the descendants of a precocious tuft progenitor cell expressing *Atoh1* transiently and/or at a low level [64]. Together these results suggest an alternative scenario taking place in the *Atoh1* ^*LoxP/LoxP*^ *; Rosa26* ^*CreERT2*^ mice in which recombination of the floxed *Atoh1* alleles would be less efficient and require higher doses of tamoxifen in progenitors than in more mature tuft lineage cells. Indeed site-specific differences in Cre recombinase expression have been demonstrated for hematopoietic organs of *Rosa26*-*CreERT2/Rosa26* mice [83]. A definite answer would require measuring the efficiency of *Atoh1* recombination in a cell-sorted population of yet unidentified tuft cell precursors.

In conclusion, although it appears that *Atoh1* expression is not needed for post-mitotic tuft cells to reach and maintain a fully differentiated state, further experiments will be needed to establish whether or not *Atoh1* function is required for tuft cell specification.

But if tuft cells share some of the pathways that govern commitment to be secretory with enteroendocrine, Paneth and goblet cells [82], there are also some differences. For instance, disruption of the Sox9 transcription factor, which is highly expressed both in tuft cells and Paneth cells, prevented the differentiation of Paneth cells [58, 60], but not that of tuft cells [34]. Similarly, deletion of the Spdef and Gfi1 transcription factors, required for differentiation of goblet and Paneth cells, did not affect tuft cell representation [34]. Finally, whereas all known enteroendocrine cell subtypes are strictly dependent on the expression of *Neurog3* [80], this is not the case of tuft cells [34], which provides support to the notion that tuft cells are not related to the enteroendocrine cell lineages (Fig. 3a, b).

So far, Gfi1b is the only transcription factor specifically expressed in tuft cells, including the crypt tuft progenitors [64]. However, taking advantage of the numerous genetically engineered mouse models already available might reveal a tuft cell-specific phenotype. The discovery of determinants of their specification and differentiation will certainly help in refining our understanding of the relationship between tuft cells and other cell types of the intestinal epithelium. The recent discovery of *Gfi1b* expression in tuft cell progenitors may be an important step towards this direction.

## Tuft cell involvement in diseases

Studies on the potential roles played by tuft cells in diseases are rare. In the airway epithelium, an involvement of tuft cells has been proposed in a patient suffering from the immotile cilia syndrome, in whom tracheal ciliated cells were absent and replaced by tuft cells [84]. Another study reported the case of an infant who developed bilateral pneumothoraces and respiratory distress soon after birth. Lung biopsies revealed desquamative interstitial pneumonitis with the presence of numerous alveolar tuft cells [85]. In this case, it was unclear whether the excess tuft cells were present prior to the disease or developed as a consequence of respiratory distress. In contrast to humans, alveolar tuft cells normally exist in the rat, and their number increase following bleomycin-induced interstitial pneumonia [86]. An increase in tuft cell representation has also been associated with gastric inflammation, hyperplasia, and metaplasia in the mouse [35]. In humans, the representation of tuft cells tends to increase in the inflamed stomach or the metaplastic intestine [35]. The situation is less clear in cancer, and although numerous cells expressing tuft cell markers, including Dclk1, can be found in mouse adenoma, they are very rare in human dysplastic lesions or colon carcinoma biopsies [34, 35]. Nevertheless, a fibrillo-caveolated carcinoma has been described, which contained cells similar to intestinal tuft cells at the morphological level [87], suggesting that tuft cells may, in rare cases, undergo transformation.

## Conclusions

The recent availability of molecular markers for tuft cells is paving the road towards the discovery of signaling molecules and transcription factors that are required for tuft cell differentiation and survival. Genetic manipulation of ex vivo organoid cultures [88], in which tuft cells differentiate from Lgr5^+^ stem cells, could greatly facilitate the identification of the pathways underlying the differentiation of the tuft cell lineage. This in turn will facilitate setting up reporter gene expression, lineage tracing experiments and gene manipulations to explore the function(s) of tuft cells in the intestinal epithelium and other epithelia, as well as their potential involvement in diseases.

## Abbreviations

Ac-tubulin: Acetylated tubulin
Atoh1: Atonal homolog 1
BrdU: 5-Bromo-2′-deoxyuridine
CFTR: Cystic fibrosis transmembrane conductance regulator homolog
CK-18: Cytokeratin 18
DCLK1: Doublecortin-like kinase 1
Dll: Delta-like
F-actin: Fibrillar actin
Gfi1: Growth factor independent 1
Gfi1b: Growth factor independent 1b
GI: Gastro-intestinal
GIP: Gastric inhibitory polypeptide
GLP-1: Glucagon-like peptide 1
Hes1: Hairy and enhancer of split 1
Hpgds: Hematopoietic prostaglandin D synthase
Klf4: Kruppel-like factor 4
L-FABP: Fatty acid-binding protein 1, liver
Lgr5: Leucine-rich repeat-containing G protein-coupled receptor 5
Neurog3: Neurogenin 3
nNOS: Nitric oxide synthase 1, neuronal
PGD2: Prostaglandin D2
PLCβ2: Phospholipase C, Beta 2
PLCγ2: Phospholipase C, Gamma 2
Ptgs: Prostaglandin-endoperoxide synthase
Sox9: SRY-box containing gene 9
Spdef: SAM pointed domain containing Rts transcription factor
T1R: Taste receptor, type 1
Trpm5: Transient receptor potential cation channel, subfamily M, member 5
UEA-1: Ulex europaeus-I lectin
